# The organization and impacts of clinical research delivery workforce redeployment during the COVID-19 pandemic: a qualitative case study of one research-intensive acute hospital trust

**DOI:** 10.1186/s12961-022-00876-5

**Published:** 2022-06-18

**Authors:** Rachel Faulkner-Gurstein, David Wyatt, Hannah Cowan, Naomi Hare, Clair Harris, Charles Wolfe

**Affiliations:** 1 King’s College London, London, UK; 2 National Institute for Health Research Biomedical Research Centre at Guy’s and St. Thomas’ NHS Foundation Trust and King’s College London, London, UK; 3 Guy’s and St. Thomas’ NHS Foundation Trust, London, UK

**Keywords:** COVID-19, Clinical research, Redeployment, Research workforce, National Health Service (UK)

## Abstract

**Background:**

COVID-19 has tested healthcare and research systems around the world, forcing the large-scale reorganization of hospitals, research infrastructure and resources. The United Kingdom has been singled out for the speed and scale of its research response. The efficiency of the United Kingdom’s research mobilization was in large part predicated on the pre-existing embeddedness of the clinical research system within the National Health Service (NHS), a public, free-at-point-of-delivery healthcare system. In this paper we discuss the redeployment of the clinical research workforce to support the pandemic clinical services, detailing the process of organizing this redeployment, as well as the impacts redeployment has had on both staff and research delivery at one research-intensive acute NHS trust in London.

**Methods:**

A social science case study of one large research-active NHS trust drawing on data from an online questionnaire; participant observation of key research planning meetings; semi-structured interviews with staff involved in research; and document analysis of emails and official national and trust communications.

**Results:**

We found that at our case-study hospital trust, the research workforce was a resource that was effectively redeployed as part of the pandemic response. Research delivery workers were redeployed to clinical roles, to COVID-related research and to work maintaining the research system during the redeployment itself. Redeployed research workers faced some difficulties with technology and communication, but many had a positive experience and saw the redeployment as a significant and valuable moment in their career.

**Conclusions:**

This study explicates the role of the research delivery workforce for the United Kingdom’s COVID response. Redeployed research workers facilitated the emergency response by delivering significant amounts of patient care. The public also benefited from having a well-developed research infrastructure in place that was able to flexibly respond to a novel virus. Many research workers feel that the NHS should provide more support for this distinctive workforce.

## Background

COVID-19 has tested healthcare and research systems around the world, forcing the large-scale reorganization of hospitals, research infrastructures and resources. The United Kingdom has been singled out by some commentators for the speed and scale of its research response, particularly regarding clinical trial setup, patient recruitment, and the delivery of globally important treatment and vaccine studies [1–8]. The efficiency of the United Kingdom’s research mobilization was in large part predicated on the pre-existing embeddedness of the clinical research system within the National Health Service (NHS), a public, free-at-point-of-delivery healthcare system. As we detail elsewhere, in addition to maintaining a routine level of service, hospitals have been tasked with delivering a high volume of emergency care alongside nationally prioritized COVID research, something which has required the large-scale reorganization of services, systems and working practices [9].

The research infrastructure in England and Wales comprises a network of institutions and organizations working to support clinical research within the NHS. While research is funded through a variety of governmental, commercial and charitable sources, the creation of the National Institute for Health and Care Research (NIHR) in 2006 was a key juncture in the development and coordination of a research system [10]. In particular, the NIHR has supported the establishment of 19 clinical research facilities (CRFs) and 20 biomedical research centres (BRCs) which provide support for experimental and translational research [3]. The NIHR also funds the Clinical Research Network (CRN), which funds and supports research staff embedded in NHS trusts across 30 different clinical specialties.

Roope et al. [11] noted that the COVID-19 pandemic has allowed us to see the “option value” of infrastructures like BRCs, CRFs and the CRN. By “option value”, Roope et al. refer to the potential value of having additional capacity that is dedicated to nonurgent work. They suggest that systems with option value are able to adapt faster and more flexibly to the needs of an unforeseen emergency, such as a global pandemic. In many ways, the United Kingdom’s research system’s response to the pandemic affirms Roope et al.’s analysis of the option value of additional capacity. But it is crucial not to lose sight of the complex reorganization of research staffing and resources that took place to facilitate the simultaneous delivery of frontline care and vital COVID research.

In this paper, we draw on original questionnaire and interview data, documentary analysis and our first-hand experience of working in a busy hospital during the first COVID surge to examine the work of research staff redeployment that allowed the United Kingdom’s research system to participate in the pandemic response. This dimension has not received the same level of public awareness as other aspects of the national and international response to COVID-19. Throughout the pandemic, the public have been informed through media reports of the stress and strain experienced by frontline care staff, with many working long hours and redeployed to different clinical areas. Clinical redeployments have also been described in several first-hand accounts (see for example [12–14]). But aside from a few exceptions in specialized professional literature (such as [14]), the patient-facing research delivery workforce has been largely absent from the discussion. This workforce includes research nurses and midwives and research allied health professionals (AHP), as well as clinical research practitioners (CRP), a newly emerging bespoke element of the research delivery workforce who do not deliver patient care outside the research pathway (for more details on the CRP role see [15]). Furthermore, accounts of research developments and breakthroughs tend to ignore the detail of how such work was delivered within these unprecedented times.

Our focus is on the redeployment of the patient-facing clinical research delivery workforce. We detail the process of organizing this redeployment, as well as the impacts redeployment has had on both staff and the clinical research delivery service at one research-intensive acute NHS foundation trust in London. We are focused specifically on three groups of research workers: those who were clinically redeployed as part of the emergency response to the pandemic, those who were redeployed to carry out urgent COVID-related research on COVID treatments and vaccines, and those who were not redeployed but were kept in place to maintain existing research.

Through our focus on the redeployment of the research delivery workforce we are able to shed light not only on the benefits and limitations of the local response to COVID-19, but also on wider questions around the existence and development of an agile, resilient and flexible research workforce, identified as a priority by the NIHR [16–18]. Our data also provide an opportunity to consider how we might change the way clinical research is organized and delivered in the United Kingdom going forward, in part due to the pace achieved in vaccine trials [19], and the change in practice this entailed. Yet, as our analysis demonstrates, while the clinical research delivery workforce are resilient and adaptable, some of the existing infrastructure and organizational practices required substantial effort to facilitate the COVID response and require further investment to underpin a system that supports agile working.

## The research delivery workforce

The research delivery system within the NHS is organized to manage, mobilize and implement clinical research protocols effectively, safely and on time [15]. Research delivery teams including nurses, midwives, AHPs and CRPs are responsible for the patient-facing aspect of clinical research, from supporting participation, undertaking informed consent, administering trial procedures and observation through to supporting governance and accurate data collection. Participating in a research delivery team requires an in-depth understanding of research protocols, the establishment of ongoing relationships with participants and principal investigators (PI), and the completion of study-specific training before trial duties are delegated.

The clinical research delivery workforce has become a significant component of the NHS. This staffing category has undergone substantial expansion in recent years, although career paths within clinical research delivery are relatively underdeveloped. Before the COVID-19 pandemic, efforts were underway to grow and professionalize this workforce with the expansion of NIHR-funded career development opportunities for research nurses and midwives and the creation of a new professional register for CRPs. COVID-19 has underscored the value of the research delivery workforce to both the United Kingdom’s COVID response and its longer-term vision of a research-led NHS [17]. Taking stock of the United Kingdom’s research response to the pandemic, health ministers from England and the devolved nations issued a white paper on the future of clinical research delivery in the United Kingdom, outlining “a bold and ambitious vision for the future of clinical research delivery, which capitalises on innovation, is resilient in the face of future healthcare challenges and improves the lives of patients around the world” [20]. Realizing this ambitious vision requires a greater understanding of the research delivery workforce and the specific skills and contributions of its workers.

## Methods

In this paper, we draw on data from a larger project which explored the impact of COVID-19 on the clinical research system and the contribution of the clinical research system to the pandemic response. Applying established social-scientific case study methods [21, 22] to one NHS trust, this project employed an online questionnaire, participant observation of key research planning meetings, semi-structured interviews with staff involved in research, and document analysis of emails and official national and trust communications. In order to maintain the anonymity of research informants, we have chosen to call our case study site South London Acute Trust (SLAT). SLAT is a research-intensive trust, hosting a number of NIHR research infrastructure installations. By December 2020, SLAT had commenced research on 80 COVID studies, with more than 18 of these classed as Urgent Public Health (UPH). UPH studies are those reviewed by the office of the Chief Medical Officer and deemed of high importance to the pandemic response. UPH studies are to be resourced and prioritized by trusts across the country [23].

We collected data from four areas within the research infrastructure: central research oversight and governance, PIs, the research delivery workforce, and patient and public involvement (PPI) managers and participants. In this paper we focus specifically on findings from an analysis of the data derived from or about the patient-facing research delivery workforce. This workforce comprises 164 staff members across the trust, working in a variety of roles, including research nurses and midwives and CRPs.

Data were collected over a 6-month period between May 2020 and October 2020. On 18 May 2020 we distributed an online questionnaire to all research-involved staff at SLAT (approximately 700) using existing mailing lists. Using the SurveyMonkey online survey platform, the questionnaire was designed as a qualitative and descriptive data-gathering tool. Questions were not drawn from a validated question bank but were reviewed and tested with a small group of research staff for clarity and comprehensiveness before wider distribution. When the questionnaire closed on 10 June 2020, we had received 170 responses, an overall response rate of approximately 24%. On first appearance, this suggests a low response rate. However, among the patient-facing research delivery workforce (*n* = 164) who are the focus of this paper, the response rate was 48% (*n* = 79), representing 46% of our total respondents. Within this group, 49% were research nurses (*n* = 39), 6% research midwives (*n* = 5) and 15% CRPs (*n* = 12). The remaining respondents were research AHPs (*n* = 6, 8%), trial coordinators (*n* = 9, 11%) or research support staff (*n* = 8, 10%). This questionnaire provided a broad overview of key issues in the organization of the research workforce during the pandemic, and specifically asked about experiences of redeployment and working during the pandemic. This questionnaire also acted as a recruitment tool for semi-structured interviews that took place later.

Interview participants were also recruited through purposive and snowball sampling, aiming to gain a sample with maximum variation [24]. Our approach aimed to gather a diversity of redeployment experiences—for example, those redeployed to frontline care, redeployed to administrative roles, redeployed to COVID research, and those who for various reasons were not redeployed. Interviewees were asked about their experiences of working during the pandemic, what their work had entailed, whether and how their work had changed because of COVID, and their views on the longer-term impact of COVID on their future work and working practices.

In total we interviewed 24 participants, of whom nine were from the research delivery workforce (see Table 1 for breakdown of research delivery workforce interviews). These nine interviews lasted between 33 and 104 minutes, averaging 45 minutes. Interviews took place over and were recorded through Microsoft Teams and were transcribed verbatim. The research delivery workers from whom we gathered either questionnaire or interview data represented the following staff groups:

**Table 1:** Breakdown of research delivery workforce study participants

	Questionnaire	Interview
Research nurse	39	4
Clinical research practitioner	12	4
Research midwife	5	
Research AHP	6	
Trial coordinator	9	1
Research support	8	
Total	79	9

Questionnaire and interview data were supplemented by an analysis of documents and emails pertaining specifically to the organization of the COVID-19 response produced by SLAT for internal use. We also analysed documents produced by national bodies (for example, the NIHR and the Chief Medical Officer for England) related to national directives on COVID-19 and clinical research. Internal documents were obtained via email from SLAT’s Research and Development (R&D) department and publicly available material on the research shutdown and UPH studies was obtained via the NIHR website. We also observed key trust research governance and prioritization meetings. These meetings, occurring twice a week, determined which COVID studies to open and discussed how studies including UPH studies were to be resourced and staffed.

Data were managed and analysed through NVivo 12 software. Data were first analysed for a descriptive account of how processes changed and how the research system and workforce were impacted [25]. Data were then analysed thematically for an analytic account [26]. RFG, HC and DW independently analysed the data and then met to agree on codes and final themes. These themes and codes were then discussed and agreed with the wider project team.

Ethical approval for this research was granted by the North East – Newcastle & Tyneside 2 Research Ethics Committee (REC) (reference: 20/NE/0138). As part of our ethics approval, we have taken steps to ensure our informants’ anonymity in the presentation of qualitative data. Interview excerpts are identified by a letter to indicate their role category and a respondent number. “D” indicates research delivery staff, “P” indicates patient and public involvement managers, and “R” indicates research leaders/PIs. “Q” denotes comments from the questionnaire.

## Results

### The process of redeployment

A reconstruction of the timeline of redeployment at SLAT suggests that while much of the process was organized quickly and reactively, with little time for detailed planning, it was successful because of the extensive, flexible, skilled workforce of research delivery staff that were in place at the trust. Redeployment drew on the specific skill set of research workers and was often crafted by them while in process. At the start of 2020, there were no redeployment guidelines, and research staff were not initially included in the trust’s emergency planning. But it immediately became clear that the defining feature of the research delivery workforce—that it operates at the nexus of care and research [15, 27]—made it a valuable resource for responding to the pandemic.

COVID-19 became a notifiable disease in March 2020, and redeployment within the NHS began soon after. On 16 March, the Department of Health and Social Care and the NIHR issued guidance stating that all clinical research conducted within NHS sites was to be halted, with specific exemptions for trials considered urgent for patient safety and COVID-related research [28]. Responding to this decree, SLAT’s R&D department asked research teams to review their portfolios and identify those activities that needed to continue and those that could be paused, alongside guidance on maintaining protocol and regulatory adherence in exceptional circumstances. For the majority of the trust’s more than 500 trials, the research shutdown meant that patient recruitment was stopped, face-to-face assessments and follow-ups were discontinued or, where possible, done remotely, and the day-to-day operation of research ground to a halt.

At SLAT, services were rapidly reorganized to meet the anticipated clinical demand, and lists were compiled of staff available to be redeployed as hospital admissions and especially admissions to the critical care and intensive care units (ICU) began to rise. At the beginning of March 2020, as the United Kingdom was entering its first lockdown, a “command and control” structure was implemented by the trust in order to centralize the pandemic response. This involved central control by the trust’s executive of daily operations at the trust following established emergency protocols. The embedded clinical research system, with its facilities, equipment and many trained staff, came to be seen as a vital resource the trust could access to bolster already stretched clinical services [9]. Though coordination with the trust’s central command structure was initially patchy, communication quickly regularized, and engagement of the clinical research delivery workforce to support the clinical response was coordinated with the research workforce lead through the trust’s nursing workforce hub.

One early hurdle to the rapid redeployment of staff was identification. Heads of nursing were required to urgently collate and submit staff skill-set and information lists so redeployment could be centrally planned and coordinated. This information did not readily exist and so necessitated collection through the circulation of spreadsheets to all the directorate research teams, which were completed manually by team managers. The process generated a real-world representation of the staffing of research teams which differed from any one centrally held electronic record.

Although the trust were trying to manage the redeployment centrally, in practice local redeployment arrangements were common. Heads of nursing made a number of informal arrangements which facilitated rapidly moving research staff into areas of need. As one senior research matron describes:

Quite a few of my team are ICU nurses. I was literally rung and said, “They’re going tomorrow. This is their last day with you. They will be gone tomorrow.” And I lost four overnight. (D-4)

By 14 April 2020, according to administrative records, there had been extensive redeployment of both clinical and nonclinical research staff. Table 2 provides a breakdown of how 152 members of the research delivery workforce were redeployed. In addition, the R&D team (covering those who work in research governance and research portfolio management) redeployed 31 staff members to act as ward clerks.

**Table 2:** Total clinical and nonclinical research staff redeployment at SLAT on 14 April 2020

Redeployed to clinical roles			Redeployed to nonclinical roles		
Role	Destination	No.	Role	Destination	No.
Adult research nurse	ICU, COVID wards, NHS Nightingale London Hospital^a^	50	Research support	Tactical subgroups	2
Paediatric research nurse	Clinical activity, NHS Nightingale London Hospital	27	Research support	Pathology/testing	7
Research midwife	Routine clinics, maternity helpline	24	Research support	Data entry	6
Clinical research practitioner	ICU turning team	14	Research support	Bereavement centre	2
Unassigned		16	Research support	Cancer centre outpatient clinics	4
Total		131	Total		21

^a^NHS Nightingale London Hospital was a temporary hospital set up by NHS England for the COVID-19 pandemic

At the same time, in March 2020, some clinical research staff were redeployed to a new COVID research team. This group comprised a variety of staff, including managers, matrons, research nurses, CRPs and data managers. This team worked to extremely tight timelines to establish treatment trials and in-house COVID research. D-1 describes some of the challenges the COVID team faced from the outset:

We’ve got a team of 22 members of staff, which have been pulled together from numerous clinical areas. Originally there was one research nurse. And that team is now a team of 22. So that’s been the biggest challenge, I suppose, is that trying to bring staff members together and quickly induct them, and again you need to get them going straightaway. You need them in and delivering on this research because we needed to start the research while we’re right in the middle of the surge in numbers. That’s been different, in that you don’t slowly take on studies. You have studies that come, they need to be set up tomorrow, recruit the first patient by the end of the week. And you need staff to deliver, even though they might be feeling wobbly in their role … and needing actually to get to grip with quite complex studies very quickly. (D-1)

Despite the challenges they faced, the group quickly developed processes for consenting highly infectious patients, established a 7-day rota and other important new procedures that required staff with knowledge of guidelines and protocols to innovate and adapt within the constraints of the regulatory framework.

As the need became more urgent and all available registered nurses were asked to go to ICU, R&D advice was strengthened and teams were asked to stop all but essential activity. The redeployment process was concluded with a final engagement of research managers to provide justification for all remaining research staff, who made up what interviewees called the “skeleton staff” for management of essential activity. Although new research activity was stopped, safety monitoring of existing participants and dispensing of trial drugs was still required, which meant some research staff were left in place to fulfil these important tasks. In small teams this was often carried out by only one person, who might now be covering several studies.

By early May the pressure of new COVID admissions on the trust was declining, and focus began shifting to gradually repatriating staff and allowing rest. At the beginning of June the plan for the restart of research was issued and the decision was taken to formally second a dedicated team to manage the ongoing portfolio of COVID research. Staff were released gradually as secondees started, with staff working on the highest-priority research being released first. The last of the redeployed clinical research staff were released by 1 September 2020.

For research delivery staff at SLAT, the pandemic and the associated shutdown of non-COVID research meant a rapid and dramatic change to “business as usual”. Asked to identify the number of studies on which they were active on 1 January 2020, 64% of respondents said they were working on five or more studies, with 37% saying they worked on nine or more studies on that date. Compare that with the figure as of 10 June—before the restart of research at the trust—of just 12% of respondents working on five or more studies, 22% saying that they were working on one study, and a further 26% working on no studies at all. However, with 74% of research delivery staff saying that they were currently working on at least one study, it is clear that while the volume of research had decreased, research activities nevertheless continued throughout the shutdown, and the research workforce remained engaged.

### Impacts of redeployment

Our data indicate that redeployment developed new skills and capacities, both for individual members of the research delivery workforce and for the research system as a whole. At the same time, it presented a number of challenges. A closer examination of some of the individual experiences of research delivery workers demonstrates both the new capacities acquired during redeployment and the challenges that this process entailed.

#### Working from home

Clinical research has hitherto been strongly tethered to clinical settings, but the response to the pandemic created capacities for new locational flexibility. As with other industries, in order to accommodate shielding and minimize the number of people in the workplace, certain forms of clinical research work moved to the home. Some research delivery staff remained in their team, but adapted to working from home, while other staff were both working from home and redeployed. What is clear is that all staff, even those who remained in post, experienced major disruptions to their work routines and to the substantive nature of the work carried out as a result of locational changes stemming from the pandemic. These disruptions reconfigured the way research was conducted at the trust, and may have far-reaching effects on the norms and processes that shape the research system into the future.

Government guidance on social distancing and avoiding unnecessary journeys preceded the official nationwide lockdown on 23 March 2020. By 17 March, research staff at SLAT were already being instructed to work from home where possible. By the time our questionnaire was conducted in June 2020, 43% of research delivery staff respondents reported that they were working from home for all or part of the week. This included staff who remained in their usual jobs but were now carrying out their work from home, as well as staff who had been redeployed to mainly administrative or clerical roles which they could perform from home. For many research delivery staff, working from home was not something routinely done before the pandemic, and it presented a set of unique challenges as well as some unexpected benefits.

A number of challenges emerged which are common to many industries that have suddenly shifted to working from home. These related to problems of technical connectivity, owing to variable internet connections or familiarity with remote working platforms. Also common to many was the problem of trying to maintain levels of productivity while not having any childcare, the feeling of working more hours, being isolated from colleagues, and not having any space away from work at home.

More specific to the work of research delivery, respondents identified issues with electronic or paper-based documentation systems that were not available from home. Accessing, storing and sending patient data is tightly controlled under the Data Protection Act 2018, and some staff found working within the guidelines complicated or even impossible to do remotely. One respondent noted:

Not all features of the clinical documentation system were accessible on my private laptop; therefore, I still had to come to SLAT sometimes to fulfil my role—even if I belong to the vulnerable group in regards to COVID. IT [information technology] services tried to help me, but I would have needed a trust laptop, which wasn't available. (Q-6)

Additionally, staff working from home had to keep up with the rapidly changing research context, which had increased the administrative burden linked to communication with colleagues—many of whom had been clinically redeployed—and patients, as well as keeping track of multiple protocol deviations and amendments.

However, for some staff the experience of working from home was not wholly negative. Some appreciated being able to remain active and contribute to the NHS’s pandemic response while shielding at home. Some appreciated the change of pace and being able to set their own schedule. One respondent suggested that “senior leadership should consider working from home as a truly viable option, rather than just a benefit to staff” (Q21), signalling the interest of some staff to continue home working after the pandemic. A shift to remote working could have wider implications for how research is organized and delivered at the trust, with the benefits of flexible hours and locations seen by some as an advantage to patients as well. As one respondent put it, “In the same vein, exploring remote options with research follow-ups would be more convenient for patients too” (Q112).

While it is too soon to say whether this shift to working from home is temporary, or what future regular home working for research delivery staff might look like, what has been made evident is that large volumes of work once performed exclusively on-site at the hospital have been transferred to remote working. This has opened up new possibilities and ways of thinking about how research delivery might be organized in the future.

#### Clinical redeployment

For many clinical research delivery staff, redeployment meant being sent to work on the clinical front line. Research nurses with ICU experience were sent to the ICU, and others were sent to work on COVID wards. Many clinical staff who were not registered nurses, including CRPs, were asked to support ICU by joining a “turning team”, a key resource-intensive intervention for the management of critically ill patients. While specific experiences varied across staff groups and locations, a common thread among our respondents was the need to quickly adapt to new ways of working in an intense clinical environment which was rapidly evolving.

Redeployment to the ICU occurred early during the first wave of the pandemic. Nurses, some of whom had not worked in ICU or on the wards in many years, found themselves thrust back to the front line. Respondents described feeling anxious about being redeployed, but also “duty-bound” (D-2) to answer the call of service. One research matron who manages a team of research nurses recounted what it was like to inform staff of their redeployment:

You had to go and talk to these people to say, “You are going back to ICU”. Now, they had all worked in ICU, but they’d left ICU because of a reason. Some of them didn’t like it. That was quite tough. You know, they were all great and they were all amazing and said, “Absolutely, we will go”. But there were lots of tears and people were scared, I think. At that point you had just heard the horror stories from Europe and everywhere and they didn’t really know what they were walking into. (D-4)

Even some nurses who had worked clinically in the more recent past felt daunted by the prospect of a return to clinical work under these conditions.

It was not just nurses who were sent to the clinical front line. CRPs who had never set foot in an ICU prior to the pandemic joined turning teams, where they were trained to turn COVID patients to the prone position, a manoeuvre which had been shown to improve respiratory failure and hypoxaemia [29]. A CRP describes her reaction to the news that she was to be redeployed to the turning team:

I’d never heard of proning before. So just went straight onto Google and Googled what proning was. And then, when we had the training session, we got to see what it actually was … I felt anxious. The weekend before the first day felt very anxious, just because it’s completely unknown to me. I’ve never set foot on an intensive care ward before, never worn PPE [personal protection equipment] before, you know. It was just all unknown. (D-5)

The result of redeployment for many staff was the opportunity to learn new skills or deepen expertise of existing ones. Of the 49 research delivery staff who answered the question on skill acquisition in the questionnaire, 59% (29) reported developing new skills through their redeployments. For some, this meant learning new computer programmes and refreshing “IT skills in use of clinical programmes that I don't need to use routinely in my research role” (Q-57). For others, redeployment meant developing new areas of clinical practice. D-4 was redeployed to a COVID research team; despite having worked in research for most of her career, she was pleased to acquire skills:

Some of the things were exciting. The first study we were doing was stem cell therapy. I’d never done that before. So, normally you probably have like 6 months to prepare for a study like that. I mean we had a week. And I hadn’t given an IV [intravenous] drug for about 10 years. The first drug I gave is stem cells. So, it was learning—it’s the steepest learning curve of my 30-year career, that first month. Every day, I had not got a clue what we were doing. (D-4)

Other interviewees also saw redeployment as potentially opening up new career trajectories:

I think there’s opportunities there for having some really good development pathways, just sort of understanding more what skills we want to develop in people. And knowing where those are amongst our teams. I think, workforce-wise, there’s loads and loads of opportunities. And really good links have been built up as well. (D-2)

For D-2, redeployment was an unprecedented opportunity for the development of new skills and career pathways.

Though redeployment was for many staff a difficult and stressful experience, many were generally positive about the opportunity to develop new skills it had provided. However, some staff were uncertain about their ability to build on these skills to advance their careers. This was particularly the case among CRPs whose uncertain career path was an issue which preceded the pandemic.

I’m a Band 6. So the next aim for me would obviously be to a Band 7. And within research, it is quite difficult once you’re at a 6 to get to a 7 because there’s minimal Band 7 roles in research. They do just get harder to progress at a certain point. So I think my trajectory is the same, because I’d probably still be in the same position without the pandemic.

*So you don’t think that the added clinical experience has made an impact on…*

No, because I’m not going to now decide to go into anything clinical. So, no, I don’t think so. The only thing it would add is, I think it does look really good on your CV [curriculum vitae] to have been involved with that. (D-5)

The experience of D-5, who had been redeployed to the turning team, illustrates a key issue for the research system. Redeployed staff acquired new skills and competencies, but since the health research system remains separate from clinical care, it is not always clear how workers will be able to apply and leverage these skills.

#### Holding the fort

While some of their colleagues were redeployed to COVID-specific roles, other redeployed staff were tasked with maintaining the research system that had been put on hold. As the pandemic continued, some research delivery staff had to remain “holding the fort” (D-2) in their research roles. Whilst there was a lighter workload as trial recruitment stopped for most studies and some questionnaire participants reported a decrease in workload, many others had to rapidly learn new skills to follow up and maintain the research process for patients already participating in trials. Some were the last member left on their team, and as such needed to both take on other colleagues’ studies and cover for those colleagues with more clinical skills who were sent to the front line. These staff who remained found an increase in stress and workload:

There has been a dramatic increase in stress, in part due to trying to keep up with ever-changing guidance. Also, as other members of the team have been redeployed and as patients feel scared, I have to work longer hours and pick up duties from where others have been redeployed, which involves learning about things I hadn't had to look at before very quickly, but am not compensated for this. (Q-7)

As one research nurse who was “holding the fort” suggested, “I’m just making sure that the patients are safe. Our main concern at the moment is, are they getting their medication, and are they safe.” (D-6).

These staff also had to quickly adapt to a number of significant procedural changes. Many patients on drug trials still had to gain access to these medications, so staff had to arrange couriers to deliver them to their doors. Scans were postponed or switched to a less time-consuming alternative where possible. Blood draws needed to be taken in health centres more local to participants to avoid travel out of the local area. Whilst some research visits still had to be conducted face-to-face, staff found switching most of their follow-ups online to be particularly challenging, especially when “dealing with anxious oncology patients, as well as my own anxieties” (Q121). Indeed, as R-5 suggested, sometimes it was “better to see patients face-to-face with particularly delicate news”, such as the discontinuation of cancer treatment.

#### Who gets to be an NHS hero?

Some redeployed research staff experienced a particular sort of stress tied to professional self-understanding and the strongly moralized narratives being promoted by the government, the media and the public at large. Despite contributing immense amounts of labour under difficult circumstances, some research delivery staff who had not been redeployed to the front line felt excluded from the “NHS heroes” public narrative which emerged early during the pandemic. Rather, as participant D-3 suggests, “There was shame and guilt if you weren’t going [to the front line]”. Other participants explained further:

I felt that, you know, really sort of useless being at home, and when there were so many brilliant colleagues that were out there really helping and getting involved right from the start. (P-1, shielded but continued work from home)

I personally volunteered for two roles but was told I couldn't because I was a nurse [needed in her team]. Instead I did nothing and felt alienated from all those who were helping out. (Q-97)

For D-3, who manages research staff, “there was very much a feeling like being the major sitting three miles back from the front line”, even though they were “currently managing everything that’s left… covering all my staff who haven’t been redeployed”. For other staff involved in clinical research, the exclusion from the “NHS heroes” narrative was underlined by the fact that they did not entirely feel like a central part of the health system even before the pandemic.

For other staff, however, the experience of redeployment to the COVID research team or the clinical front line contributed to a strong feeling of pride at having been part of the NHS pandemic response, alongside clinical colleagues. Many respondents, especially CRPs and those whose work was typically confined to the spaces and routines of research, described a new sense of belonging within the trust.

I felt really privileged actually to be able to be there. You know, we were chatting and, in 20 years, when you look back and you think, what did you do in COVID, I would definitely say I was at the forefront doing research, which is lovely. (D-4)

The presence of research nurses, CRPs and other research staff on COVID wards and the high profile of the COVID research being undertaken at the hospital contributed to many clinical research staff feeling visible and appreciated in the eyes of colleagues, if not also by the wider public.

I think it’s been really good for research because it’s really raised the profile that research nurses and practitioners are clinical, patient-facing, and they have clinical skills and expertise to be able to meet the need of clinical areas during this pandemic. And that kind of flexibility and willingness of us to kind of have released staff and tried to release them quite quickly in response to that appropriately. (D-1)

Although the “NHS heroes” narrative emphasized the role of frontline care workers, others recognized that there were many different ways for research staff to contribute to supporting the health system during this exceptional time.

## Discussion and conclusion

This study has examined redeployment of the research delivery workforce in one research-active NHS trust during the pandemic. Drawing on empirical data collected between May 2020 and October 2020 as well as wider documentary analysis, we have demonstrated that at SLAT, the research workforce was able to function as a resource that was effectively redeployed as part of the pandemic response. Redeployment was in many ways an improvised process, but it drew on the specific skill set and professional profile of the research delivery workforce. It presented some unique challenges for research workers but also created new capacities for the research system. Redeployed research workers faced some difficulties with technology, communication and stress, but many also had a positive experience and saw the redeployment as a significant and valuable moment in their career and a way to contribute to the national pandemic response.

Although one case study of one trust cannot yield data generalizable to all trusts, the validity of these findings is supported by the broad and varied qualitative data set upon which it is based. This study benefited from first-hand operational experience of the redeployment process, as well as multiple forms of qualitative data. Our broad sample of the research delivery workforce at SLAT allowed us to gain in-depth perspective on the pandemic response within the trust’s clinical research system.

At the same time, we recognize that this study has a number of limitations. Our sample was designed to be illustrative of a significant case, not representative of all cases. The location of the trust in central London, the size of the research workforce and the scale of research activity mean that our case is unlikely to be perfectly representative of trusts in other areas or with varying levels of research activity. Additionally, data collection was prospective rather than retrospective—data collection took place while the pandemic was ongoing. This meant that some day-to-day concerns may have been amplified. Seen retrospectively, our study participants might report a different set of experiences.

This study suggests a number of ways that, going forward, trusts might better draw upon and support their research workforces. One of the clearest lessons to be learned from this case is that the relationship between research and clinical care can be clarified and further developed. At SLAT, large amounts of research are carried out, but research is not as well integrated with clinical functions as it could be. During redeployment, this was reflected in some awkward initial stages where it was not clear what role research delivery workers would play. Our findings also suggest that trusts should start to think more holistically about research not as an exceptional activity but as part of the business as usual of the trust. Researchers constitute a trained, flexible workforce embedded in trust core function, supporting patients who choose research as part of their care pathway. The process of redeployment at SLAT provides support for Roope et al.’s “option value” argument [11]: the research delivery workforce did not represent unnecessary excess capacity but rather an agile, flexible and valuable resource that was able to mobilize effectively in an unprecedented emergency. Our study also speaks to the wider importance of an integrated and agile research infrastructure that sits outside of individual research projects. Zakaria et al. [30] discuss some of the key challenges of assessing the impact of research infrastructures. Acknowledging how impact assessment can often be focused on discrete research projects, Zakaria et al. call for debate in how we evaluate the impact of research infrastructures specifically. While our case study does not provide a framework to measure impact, it does demonstrate some of the key benefits and impacts of research infrastructures that may be overlooked in current impact assessment practices—in particular, the skills and adaptability of the research delivery workforce, the “option value” offered by both the research delivery workforce and the research infrastructure more broadly, which allows the system to support pressing and emerging research needs across the whole research portfolio, and support care delivery in emergencies.

In the exceptional circumstances of the COVID-19 pandemic, the research delivery workforce provided much-needed support to the NHS, but particularly in an NHS that is pushing to develop its research capacity and instil research into everyday NHS activity, using the skills and adaptability of the research infrastructure more broadly is not a solution to everyday staffing problems which span well beyond the confines of individual NHS trusts. Even in the case discussed here, the research delivery workforce and wider research infrastructure (including the R&D department) represent just 2% of the trust’s staff numbers.

Our case also suggests a number of specific ways that the research workforce could be supported. For example, during redeployment, CRPs ended up performing important clinical patient-facing roles. Recognizing this, trusts might seek to supplement their skills base, in order to make their skills more visible to supervisors and uniform across departments. Trusts might also engage in other forms of upskilling for research delivery workers. Many research staff appreciated the opportunity that redeployment gave them to learn new skills, interact with new colleagues and patients, and participate in new parts of the trust. After the pandemic, more avenues for skill acquisition and lateral working should be offered. This would build on the linkages between the often-siloed research system and the day-to-day clinical services at the trust and could be a benefit to both, and ultimately to patients.

At the same time, many research delivery workers, like their colleagues across the health service, experienced burnout, trauma and exhaustion. For some research delivery workers, this was exacerbated by the knowledge that the opportunities to build upon new skills acquired during redeployment are limited. Trusts could help support the research workforce by clarifying their career trajectories and ensuring that there are adequate forms of advancement and progression available to them. Work is being done here at a national level through the NIHR to develop the profession and professional identity of the CRP [31]. However, the pandemic has rendered issues around skills development and documentation and advancement within this workforce into sharper focus.

Above all, this study demonstrates the “option value” and potential of the research delivery workforce. Patients benefited from the care that redeployed research workers were able to offer. The public benefited from having a well-developed research infrastructure that could facilitate rapid respond to a novel virus. The NHS should recognize the value of this workforce and support it accordingly.

The pandemic represented a high-stakes stress test for the United Kingdom’s research infrastructure and the workforce that keeps it running. Our case suggests that this workforce successfully delivered crucial research as well as vital clinical support during a critical time. The health service and the British public only stand to benefit by investing in the development of and support for these unique medical workers.

## Data Availability

The data generated and analysed during the current study are not publicly available, as they contain information that could compromise research participant privacy, but are available from the corresponding author on reasonable request.
